# *yaaJ*, the tRNA-Specific Adenosine Deaminase, Is Dispensable in *Bacillus subtilis*

**DOI:** 10.3390/genes14081515

**Published:** 2023-07-25

**Authors:** Akiko Soma, Atsushi Kubota, Daisuke Tomoe, Yoshiho Ikeuchi, Fujio Kawamura, Hijiri Arimoto, Yuh Shiwa, Yu Kanesaki, Hideaki Nanamiya, Hirofumi Yoshikawa, Tsutomu Suzuki, Yasuhiko Sekine

**Affiliations:** 1 Graduate School of Horticulture, Chiba University, 648 Matsudo, Chiba 271-8510, Japan; 2 Department of Life Science, College of Science, Rikkyo University, 3-34-1 Nishi-Ikebukuro, Toshima-ku, Tokyo 171-8501, Japan; 3 Department of Chemistry and Biotechnology, Graduate School of Engineering, The University of Tokyo, 7-3-1, Hongo, Bunkyo-ku, Tokyo 113-8656, Japan; 4 NODAI Genome Research Center, Tokyo University of Agriculture, Tokyo 156-8502, Japan; 5 Department of Molecular Microbiology, Tokyo University of Agriculture, Tokyo 156-8502, Japan; 6 Shizuoka Instrumental Analysis Center, Shizuoka University, 836 Ohya, Suruga-ku, Shizuoka 422-8529, Japan; 7 Fukushima Translational Research Foundation, Capital Front Bldg., 7-4, 1-35, Sakae-machi, Fukushima 960-8031, Japan; 8 Department of Bioscience, Tokyo University of Agriculture, 1-1-1 Sakuragaoka, Setagaya-ku, Tokyo 156-8502, Japan

**Keywords:** tRNA, post-transcriptional modification, inosine, four-way wobbling, *Bacillus subtilis*

## Abstract

Post-transcriptional modifications of tRNA are crucial for their core function. The inosine (I; 6-deaminated adenosine) at the first position in the anticodon of tRNA^Arg^(ICG) modulates the decoding capability and is generally considered essential for reading CGU, CGC, and CGA codons in eubacteria. We report here that the *Bacillus subtilis yaaJ* gene encodes tRNA-specific adenosine deaminase and is non-essential for viability. A β−galactosidase reporter assay revealed that the translational activity of CGN codons was not impaired in the *yaaJ*-deletion mutant. Furthermore, tRNA^Arg^(CCG) responsible for decoding the CGG codon was dispensable, even in the presence or absence of *yaaJ*. These results strongly suggest that tRNA^Arg^ with either the anticodon ICG or ACG has an intrinsic ability to recognize all four CGN codons, providing a fundamental concept of non-canonical wobbling mediated by adenosine and inosine nucleotides in the anticodon. This is the first example of the four-way wobbling by inosine nucleotide in bacterial cells. On the other hand, the absence of inosine modification induced +1 frameshifting, especially at the CGA codon. Additionally, the *yaaJ* deletion affected growth and competency. Therefore, the inosine modification is beneficial for translational fidelity and proper growth-phase control, and that is why *yaaJ* has been actually conserved in *B. subtilis*.

## 1. Introduction

**Figure 1:**
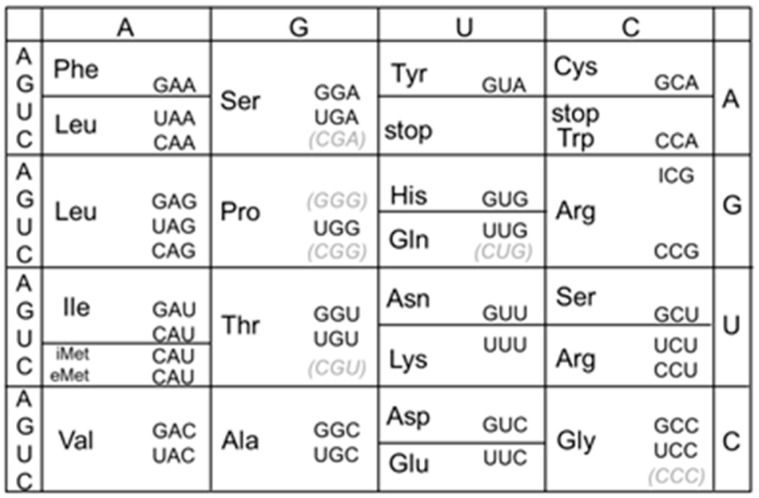
Anticodons in *B. subtilis* and *E. coli*. All anticodons are shown in the 5′ to 3′ direction. Modified nucleotides other than the inosine of tRNA^Arg^ are omitted. iMet and eMet indicate initiator and elongator tRNA^Met^(CAU), respectively. The anticodon CAU responsible for isoleucine is modified to LAU (L; lysidine) in *B. subtilis* and *E. coli* [5,6,7]. Anticodons that are not found in *B. subtilis* but occur in *E. coli* are in parentheses [11,18].

**Figure 2:**
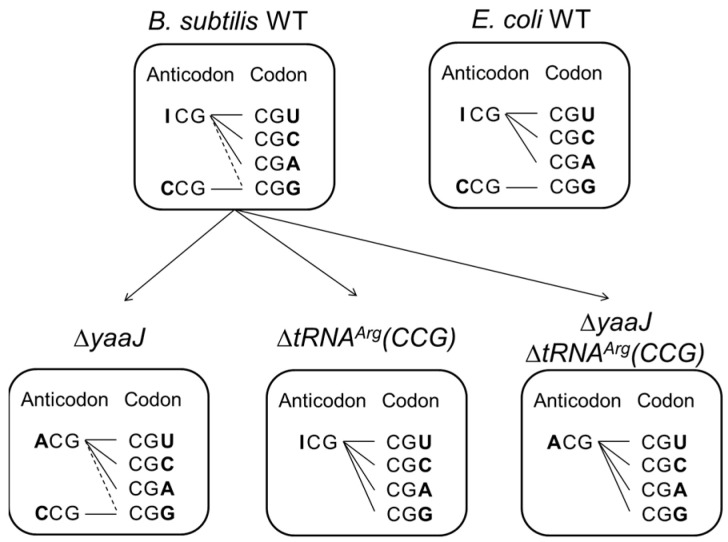
Decoding model of CGN codon family in *B. subtilis* and *E. coli*. All codons and anticodons are shown in the 5′ to 3′ direction. For most eubacteria, tRNA^Arg^(ICG) dominantly decodes three arginine codons, namely CGU, CGC, and CGA, whereas tRNA^Arg^(CCG) decodes the CGG codon. In *B. subtilis* and *E. coli*, tRNA^Arg^(ACG) and tRNA^Arg^(CCG) are encoded by four and a single gene, respectively. The genes encoding tRNA^Arg^(ACG), tRNA^Arg^(CCG), and tRNA adenosine deaminase (TadA) are all essential for the viability of *E. coli*. In *B. subtilis*, as indicated by dashed lines, CGG codons may be redundantly recognized by two tRNAs, namely tRNA^Arg^(ICG) and tRNA^Arg^(CCG) in wild-type cells (WT), or by tRNA^Arg^(ACG) and tRNA^Arg^(CCG) in the *yaaJ*-deletion mutant cells (Δ*yaaJ*). All four CGN codons can be decoded by either (1) tRNA^Arg^(ICG) in the *trnQ-Arg(CCG)*-deletion mutant (Δ*tRNA^Arg^(CCG)*), or by (2) tRNA^Arg^(ACG) in the double-deletion mutant of *yaaJ* and *trnQ*-Arg(CCG) (Δ*yaaJ*Δ*tRNA^Arg^(CCG)*).

A productive codon–anticodon pairing on the ribosome is crucial for efficient and accurate protein synthesis [1,2,3,4]. Post-transcriptional modifications of tRNA molecules are functionally important, and nucleotide modification at the wobble position of the anticodon (position 34) plays a key role in codon recognition [5,6,7,8]. Inosine (denoted as I) is the 6-deaminated form of adenosine, and in this form, its chemical structure is similar to guanosine because the amino group (hydrogen donor) at position 6 is substituted for the keto group (hydrogen acceptor) [9,10]. In eubacteria, inosine modification of RNA molecules is usually identified at the wobble position of the anticodon of tRNA^Arg^(ICG), which is one of the tRNA repertoires responsible for decoding the CGN four-codon family (Figure 1) [6,7,10,11,12,13]. Well-studied eubacteria, including *Escherichia coli* and *B. subtilis*, contain two species of tRNA for decoding CGN codons. According to the orthodox base-pairing theory, it is generally assumed that tRNA^Arg^(ICG) recognizes CGU, CGC, and CGA codons, whereas tRNA^Arg^(CCG) recognizes the CGG codon (Figure 2, upper right) [13,14,15,16,17,18].

Base pairings between unmodified A34 and N(III) (where N denotes U, C, A, or G at the third position of the codon) are theoretically possible, though they are unstable or nonproductive, and sequencing analyses of tRNA gene sets from various organisms have revealed deductively that unmodified A34 can recognize all four nucleotides at the wobble position of the codon [11,13,14,19,20,21,22,23,24,25,26,27,28,29]. Pairings between A34 and R(III) (where R denotes a purine nucleotide) are particularly unstable, and they have been predicted to form only with non-canonical conformations of nucleotides. As for A34-Y(III) pairings (where Y denotes a pyrimidine nucleotide), in vitro and in vivo analyses have shown their instability and decoding inefficiency. Furthermore, it has been reported that an unmodified A34 on the ribosomal P site destabilizes the next A site codon–anticodon duplex during translation [22], suggesting that unmodified A34 is not preferred for decoding. Inosine modification would alleviate such adverse effects of A34 and modulate its reading of codons. A34 is post-transcriptionally converted to I34 in nature, and unmodified A34 in mature tRNAs has been found in some mycoplasmas and organelles that contain compact genomes encoding a reduced set of tRNA repertoires [11,30,31,32].

Crystallization of the 30S subunit of the *Thermus thermophilus* ribosome complexed with an anticodon stem-loop oligonucleotide containing inosine at the wobble position of the anticodon has confirmed the presence of two hydrogen bonds in the I34-A(III) pairing with the anti-conformation geometry of sugars, as proposed by Crick [1,15,24]. In this context, it is generally thought that I34 modification is crucial for decoding three codons by a single species of tRNA^Arg^ and that this modification has evolved as a solution to decoding the A-ending codons [5,6,7,10,13].

The pairing of the ICG anticodon with the CGG codon does not occur because the I34-G(III) pairing likely causes steric hindrance, although it is possible only when it involves unusual tautomerization or geometry of nucleotides under specific conditions [13,24,27,28,29]. Thus, tRNA^Arg^(CCG) should be required for decoding CGG codons (Figure 2), and both tRNA^Arg^(ICG) and tRNA^Arg^(CCG) have been shown indispensable for the viability of *E. coli* [33].

Inosine formation generally occurs through hydrolytic deamination of genomically encoded adenosine, a process that is catalyzed by adenosine deaminase [9,10,17]. Inosine synthase in the *E. coli* K12 strain was identified as the first prokaryotic tRNA-specific adenosine deaminase (TadA) [34]. It has been shown that *tadA* is broadly conserved among eubacteria with exceptions in some mollicutes [31]. The deletion mutant of *E. coli tadA* could not be obtained using the direct gene disruption method [34], supporting the early prediction that I34 is crucial for translation in eubacteria. In contrast, we found that *yaaJ*, the *B. subtilis* homolog of *tadA*, does not appear on published lists of *B. subtilis* essential genes [10,35,36,37], implying that the I34 modification of tRNA^Arg^(ACG) is not essential for viability. This observation encouraged us to elucidate the function of the inosine modification of tRNA^Arg^(ICG) and the decoding strategy of the CGN codon family in *B. subtilis*.

Here, we show that *yaaJ* is the only gene responsible for the I34 formation of tRNA^Arg^ and that it is completely dispensable for the growth and translation in *B. subtilis*. We also found that tRNA^Arg^(CCG) responsible for the CGG codon is non-essential both in the presence and absence of *yaaJ*. These findings demonstrate the decoding of all four CGN codons by a single species of tRNA involving non-canonical A34-N(III) and I34-N(III) pairings; this is the first example of non-discriminating four-way wobbling by inosine nucleotide in bacterial cells. On the other hand, the *yaaJ* deletion caused a marked increase in frameshifting at the CGA codon, indicating that I34 contributes to translational fidelity. Additionally, the growth of the *yaaJ*-deletion mutant was affected in the poor medium at higher temperatures and was significantly impaired in natural competence. These results suggest the physiological importance of I34 in stress responses.

## 2. Materials and Methods

- Construction of *B. subtilis* strains of the *yaaJ* and tRNA^Arg^(CCG)

Deletion mutants of *yaaJ* and tRNA^Arg^(CCG) listed in Table 1 were constructed by replacement of each gene in the genome of the *B. subtilis* 168 strain with a PCR fragment that was designed to contain the antibiotic-resistance gene flanked by the target gene region (Figure S1) and generated using the primers listed in Table S1. In all cases, the correct introduction of the deletion mutation was confirmed by back-crossing into strain 168, followed by PCR and DNA sequencing as a second verification.

- tRNA isolation

Total RNA was prepared from late-log phase *B. subtilis* cells cultured in LB at 37 °C, using ISOGEN (Nippon Gene, Toyama, Toyama, Japan), as described previously [38]. Then, crude tRNA was abstracted from the total RNA by fractionation on a Bio-scale DEAE2 column (Bio-Rad, Hercules, CA, USA) with a linear gradient of NaCl consisting of solvent A (200 mM NaCl, 20 mM Hepes-KOH [pH 7.5] and 8 mM MgCl_2_) and solvent B (700 mM NaCl, 20 mM Hepes-KOH [pH 7.5] and 8 mM MgCl_2_) at a flow rate of 2 mL/min. tRNA^Arg^(I/ACG) was isolated from crude tRNA according to the solid-phase DNA probe method described previously [39] using the 3′-biotinylated probe (Table S1). Denaturing PAGE was used for further purification of the isolated tRNA^Arg^ molecule.

- Mass spectrometry

The isolated RNAs were digested into nucleosides and analyzed by LC/MS using ion-trap mass spectrometry as described previously [40], with the following slight modifications. An LCQDUO ion-trap (IT) mass spectrometer (Thermo Fisher Scientific, Waltham, MA, USA) equipped with an electrospray ionization (ESI) source and HP1100 liquid chromatography system (Agilent Technologies, Santa Clara, CA, USA) were used to analyze the nucleosides. The isolated tRNA^Arg^(I/ACG) was digested with P1 nuclease (Yamasa, Noda, Chiba, Japan) and alkaline phosphatase (*E. coli* C75, Takara, Kusatsu, Shiga, Japan) in a 25 μL reaction mixture containing 20 mM HEPES-KOH (pH 7.6) at 37 °C for 3 h and then analyzed by LC/MS. The hydrolysate was fractionated by using an Inertsil ODS-3 column, 250 × 2.1 mm (GL Science, Shinjuku-ku, Tokyo, Japan). The solvent system consisted of 5 mM NH_4_OAc (pH 5.3) (A) and 60% acetonitrile (B), used as follows: 1–35% B in 0–35 min, 35–99% B in 35–40 min, and 99% B in 40–50 min. The chromatographic effluent (150 μL/min) was directly conducted into the ion source without prior splitting. Positive ions were scanned over an *m*/*z* range of 103–700 throughout the separation under the following conditions: flow rate of sheath gas, 95 arb; capillary temperature, 245 °C; and spray voltage, 5 kV.

- Sporulation assay

*B. subtilis* cells were inoculated at OD_600_ of ca. 0.03–0.05 and grown in 2× SG medium (2× Schaeffer’s sporulation medium supplemented with 0.1% glucose, [41]) for 24 h at 37 °C with shaking. To assay for cells that had produced heat-resistant spores, the culture was heated at 80 °C for 10 min, plated on LB agar plates, and incubated at 37 °C for 24 h. The number of cell colonies, both those subjected and not subjected to heat treatment, was then compared.

- Transformation efficiency assay

Trp+ transformation activity of strains was determined using 168W (*trp+*) chromosomal DNA. Cells were grown in a competence-inducing (CI) medium consisting of Spizizen’s minimal glucose (0.5%) (MMG) medium supplemented with 0.03% yeast extract instead of casein acid hydrolysate [42] at 37 °C with shaking until the OD_600_ reached 1.0. An aliquot (0.1 mL) of the culture was withdrawn and mixed with the DNA solution at a final concentration of 2 μg ml^−1^. After incubation for 30 min at 37 °C with shaking, the cells were plated on MMG agar plates. Trp+ transformants were counted after 2 days of incubation at 37 °C.

- Northern blot analysis of *B. subtilis* tRNA^Arg^(I/ACG) and tRNA^Arg^(CCG)

Total RNA prepared from late-log phase *B. subtilis* cells cultured in LB at 37 °C, using ISOGEN (Nippon Gene, Toyama, Toyama, Japan), was separated on an 8% polyacrylamide gel containing 8 M urea. The gel was blotted onto Hybond N+ (GE Healthcare Bio-science AB, Uppsala, Sweden) for Northern blotting analysis. Hybridization was performed by using 5′ ^32^P-labeled synthetic DNA oligonucleotides (Table S1) as probes [38].

- Reverse-transcription polymerase chain reaction (RT-PCR) and DNA sequencing

RT-PCR and sequencing analysis followed the previous study [43]. Total RNA prepared from *B. subtilis* cells as described above was reverse-transcribed with ReverTraAce (TOYOBO, Osaka, Osaka, Japan) and PCR-amplified with Blend Taq (TOYOBO, Osaka, Osaka, Japan). Pimers used for RT-PCR are listed in Table S1. PCR products were gel purified and cloned using the TA cloning kit (Invitrogen, Carlsbad, CA, USA) according to the manufacturer’s protocol. Between 50 and 100 clones were sequenced for each product.

- β−Galactosidase assay

The *lacZ*-coding region of pMC1871 [44] was transferred to pAPNC213 [45] to construct the reporter plasmid pTOM20c, as described previously [38]. The peptide sequence DERRKLRR, which is encoded by two sets of tandem CGN (i.e., CGU, CGC, CGA, CGG, or AGA) codons, was inserted between the first and second amino acid residues of *lacZ* in pTOM20c to construct the plasmid pTOM21, pTOM22, pTOM23, pTOM24, and pTOM25, respectively. pTOM26 encoding the peptide sequence DEAAKLAA was also used as a control. Synthetic DNA oligonucleotides used for plasmid constructions are listed in Table S1. The *B. subtilis* strains transformed with these plasmid DNA linearized by *Sca*I digestion to integrate the *lacZ* gene into the *aprE* site of the genome of the wild-type or the deletion mutants of *yaaJ* and *trnQ-Arg(CCG)* were used for the β−gal assay (KUB11-32 in Table S2). Precultures of each strain were inoculated into fresh LB media with 1 mM IPTG and cultured at 37 °C until the OD_600_ reached 0.6. The β−gal assay was performed according to the references [38,46].

- Frameshift assay

Our methods were based on previous studies of the *E. coli* release factor 2 (RF-2) programmed frameshift mechanism [47,48]. Plasmids encoding *lacZ*, which contains the CGU, CGC, CGA, CGG, or UGA codon at the frameshifting regulatory site at the N-terminus of *lacZ* were constructed and designated pKUB20, pKUB21, pKUB22, pKUB23, or pKUB24, respectively. As a control, pKUB25 without the frameshifting regulatory site was used. Synthetic DNA oligonucleotides used for plasmid constructions are listed in Table S1. The *B. subtilis* strains transformed with these plasmids to integrate the *lacZ* gene into the *aprE* site of the genome, as described above, were used for β−gal assay (KUB33-44 in Table S2).

- Quantitative real-time PCR (qPCR)

*B. subtilis* cells grown in LB at 37 °C were harvested when the OD_600_ reached 0.6; total RNA was prepared using RNeasy Mini Kit (Qiagen, Hilden, Germany) according to the manufacturer’s protocol. Next, cDNA was prepared from total RNA by reverse transcription using random hexamer primer and ReverTraAce (TOYOBO, Osaka, Osaka, Japan) according to the manufacturer’s instructions. Expression of the target genes was determined by qPCR, using the primers listed in Table S1, with Power SYBR Green PCR Master Mix and a 7500 Real-Time PCR System (Applied Biosystems, University Park, IL, USA). Transcript abundance was normalized to 16S rRNA. The qPCR reactions were carried out using RNA isolated from at least three independent cultures.

## 3. Results

- *B. subtilis yaaJ* encodes a tRNA adenosine deaminase and is dispensable but beneficial for growth and competency

**Figure 3:**
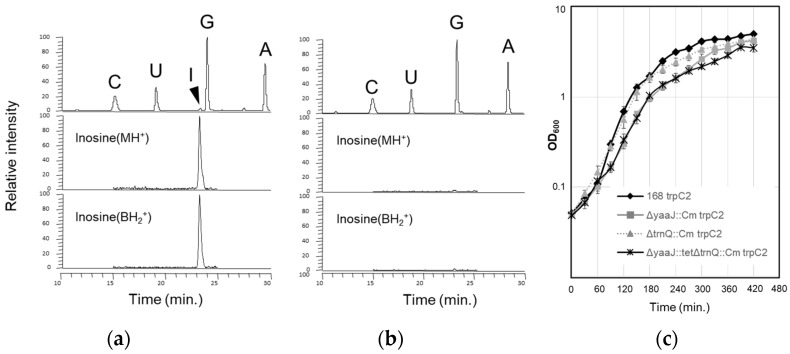
Mass spectrometric analyses of tRNA^Arg^(I/ACG) and growth of *B. subtilis* strains. LC/MS nucleoside analysis of purified tRNA^Arg^(I/ACG) from (**a**) the wild-type (strain 168) and (**b**) KUB10 strains. The upper panel shows the UV trace at 254 nm, and the position of each nucleoside is indicated. The middle and lower panels show mass chromatograms detecting proton adduct (MH+, *m*/*z* 269) and the base-related ion (BH_2_+, *m*/*z* 137) of inosine, respectively. (**c**) Growth of the wild-type (strain 168; diamonds with black line), *yaaJ*-deletion mutant (KUB10; squares with gray line), *trnQ-Arg(CCG)*-deletion mutant (SOM1; triangles with gray dashed line), and a double deletion mutant of *trnQ-Arg(CCG)* and *yaaJ* (SOM2; crosses with black line) strains in LB medium at 37 °C with shaking. Data are represented as mean ± SD, *n* = 3.

*B. subtilis* YaaJ, a homolog of *E. coli* TadA, possibly encodes a tRNA-specific adenosine deaminase [34,35,36]. The deletion mutant of *yaaJ* has been constructed by the *B. subtilis* genome project [36], suggesting that *yaaJ* is non-essential for viability. To confirm that *yaaJ* is completely dispensable and responsible for inosine formation, a deletion mutant strain (KUB10) was constructed by replacing the coding region of *yaaJ* with a gene encoding chloramphenicol acetyltransferase (Figure S1a). The genes located downstream of *yaaJ* (*scr-dnaX-yaaK-recR-yaaL-bofA*) harbor their own promoters, and thus the deletion of *yaaJ* does not inhibit the expression of the downstream genes. Next, the solid-phase DNA probe method [39] was used to isolate tRNA^Arg^(ICG) or tRNA^Arg^(ACG) from wild-type (strain 168) and KUB10 cells cultured in LB medium at 37 °C (a typical laboratory growth condition); inosine formation of tRNA^Arg^ was analyzed by mass spectrometry (Figure 3a,b). Analysis of the total nucleosides derived from the digested tRNA^Arg^ samples revealed the specific disappearance of inosine from the KUB10 cells, indicating that *yaaJ* is required for the inosine modification of tRNA^Arg^. The sequence of the anticodon was confirmed by a reverse transcription polymerase chain reaction (RT-PCR) of tRNA^Arg^(I/ACG) followed by a sequencing analysis. Since inosine is recognized as guanosine by reverse transcriptase, “I” was represented as “G” at the corresponding position in the sequencing outputs. The results revealed that tRNA^Arg^ from the KUB10 cells harbored the anticodon sequence ACG (Figure S2b), whereas those from the wild-type harbored the anticodon sequence GCG only (Figure S2a). Northern blotting showed that tRNA^Arg^ with unmodified A34 is as stable as that with I34 in wild-type cells (Figure S3).

Growth of KUB10 strain in LB medium at 37 °C was slowed during the log phase (Figure 3c), and such a tendency was even clearer when grown at 45 °C (Figure S4a). This observation seems to be due to the limiting effect of increased temperature on the KUB10 growth rate. The growth rate of the wild-type strain increased with the temperature both in rich (LB, Figure S4a) and poor media (CSM; competence and sporulation medium, Figure S4b). However, this was not observed for KUB10. It was intriguing that the growth curves of KUB10 in CSM were almost the same at 37 °C and 45 °C (Figure S4b).

*B. subtilis* cells respond to a range of stress by spore formation. This process involves up to 500 genes [49,50,51,52,53] that tend to use minor codons including the CGA codon [54,55]. We then examined the effect of *yaaJ* deletion on heat-resistant spore formation. KUB10 showed only a slight decrease in sporulation efficiency (Table 2) suggesting that inosine modification of tRNA^Arg^ is not essential for the expression of sporulation genes. On the path toward sporulation, some of the *B. subtilis* cells become naturally competent and thus have the opportunity to adapt to their environment [51,53]. We found that the competency of KUB10 decreased by 100-fold (Table 3), indicating the significance of the *yaaJ* gene. The development of competency in *B. subtilis* is regulated by a complex and sophisticated signal transduction network. Expression of the competence genes that mediate DNA uptake and recombination are driven mostly by a master regulator comK during transformation. Then, we performed a reporter assay to monitor the expression of *comK*. We also checked the expression of *srfA*, which is located upstream of *comK* in the regulatory cascade and positively regulates the stability of the comK protein. The translational fusion of the regulatory region and the first six codons (not arginine codons) of *comK* or *srfA* to the N-terminus of *lacZ* [56] was integrated into the *amyE* site of the wild-type or KUB10 genome, yielding strains that express β−gal under the control of *comK* or *srfA* regulatory sequences. When the KUB10 strains harboring *comK-lacZ* (Figure S5, middle) or *srfA-lacZ* (Figure S5, top) were grown on the CI plate medium (with X−gal) which induces competence, they turned white or blue, respectively. Therefore, the inhibitory effect of the *yaaJ*-deletion seems attributable to the defect of the comK synthesis. To understand the detailed mechanism for this phenomenon, further analysis will be required.

Based on these observations, we concluded that (1) *B. subtilis yaaJ* encodes a tRNA-specific adenosine deaminase, (2) inosine formation at the wobble position of tRNA^Arg^(ICG) is dispensable yet beneficial for the growth, and (3) *yaaJ* is important for the development of competency.

- Translational activity without I34 of *B. subtilis* tRNA^Arg^

**Figure 4:**
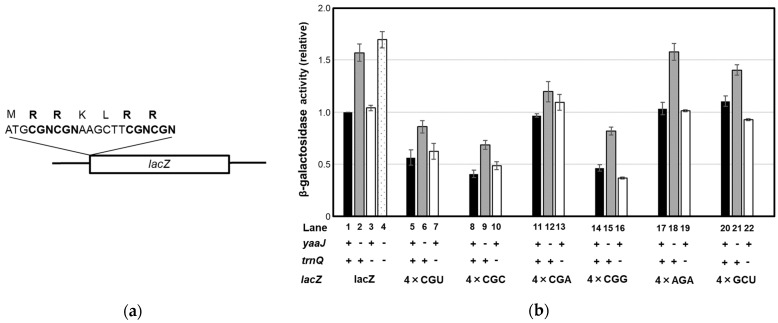
β−galactosidase assay with or without I34 modification and tRNA^Arg^(CCG). (**a**) Schematic illustration of the *lacZ* reporter genes used in the β−gal assays, showing the inserted nucleotide and peptide sequences. The two tandem arginine (R) residues encoded by the CGN codons in the inserted peptide are indicated in bold. Negative controls were also used in which the CGN codons are substituted by AGA (non-CGN arginine codon) or GCU for alanine (non-arginine codon). (**b**) β−gal activity of the unmodified *lacZ*, 4 × CGN-*lacZ*, 4 × AGA-*lacZ,* or 4 × GCU-*lacZ* reporter genes in *B. subtilis* strains with (+) or without (−) *yaaJ* and *trnQ*-*Arg(CCG)*.

The results described above raised the question of how the CGN codon family, particularly CGC and CGA codons, are decoded by tRNA^Arg^ without I34 in *yaaJ*-deletion mutant cells. Decoding both CGC and CGA codons by tRNA^Arg^(CCG), another repertoire for the CGN codon box is generally implausible. Alternatively, tRNAs responsible for amino acids other than arginine may recognize those codons resulting in mistranslation. However, CGC and CGA codons are frequently used in essential genes in the *B. subtilis* genome: genes for DNA polymerases (e.g., *polC* and *dnaE*), RNA polymerases (e.g., *rpoA* and *rpoB*), aminoacyl-tRNA synthetases (e.g., *metS* and *argS*), and ribosomal proteins (e.g., *rplS*, and *rpsO*) [35,36,55]. Therefore, in KUB10 cells, tRNA^Arg^(ACG) acting cooperatively with tRNA^Arg^(CCG) is expected to translate all CGN codons mostly as arginine. To analyze the fundamental decoding property of CGN codons in *B. subtilis*, a reporter assay was performed using the β−gal, which originally contains 20, 36, 3, and 7 CGT, CGC, CGA, and CGG codons, respectively. We also used the *lacZ* derivatives (4 × CGN−*lacZ*) where two pairs of tandem CGN codons, which are intervened by two non-arginine codons, are fused to the N-terminus of *lacZ* (Figure 4a). Each *lacZ* gene was integrated into the *amyE* locus of the wild-type and KUB10 genomes (Table S2). In the wild-type strain, the β−gal activity of the original *lacZ* and the 4 × CGA−*lacZ* was comparable to each other (lanes 1 and 11 in Figure 4b), showing that continual CGA codons do not impede translation in *B. subtilis* cells. In KUB10, the β−gal activity of the original and *lacZ* derivatives was not impaired but rather higher (lanes 2, 6, 9, 12, 15, 18, and 21 in Figure 4b) than that in the wild-type strain (lanes 1, 5, 8, 11, 14, 17, and 20). The increase of β−gal activity was smallest when the 4 × CGA−*lacZ* was used (lanes 11 and 20), suggesting that a tract of CGA codons affects the production of β−gal protein in KUB10 cells. Quantitative real-time PCR analysis of *lacZ*−mRNAs showed that the amount of mRNA of the original *lacZ* in KUB10 cells is higher (lanes 1 and 2 in Figure S6), while that of the 4 × CGA−*lacZ* is almost the same as the wild-type strain (lanes 3 and 4 in Figure S6). Therefore, the small increase of β−gal activity of the 4 × CGA−*lacZ* in the absence of I34 was possibly derived from the reduced amount of mRNA. The exact mechanism for the increases in the β−gal activity is unclear. Translational activity sometimes affects mRNA stability [57,58,59], and thus active translation caused close spacing of translating ribosomes on the β−gal mRNA to protect mRNA from cleavage. It is also possible that the slower translation in the absence of *yaaJ* rather stabilized the *lacZ*−mRNA and resulted in a higher expression of the β−galactosidase protein.

- Lack of I34 increases the rate of frameshifting at the CGA and CGC codons

**Figure 5:**
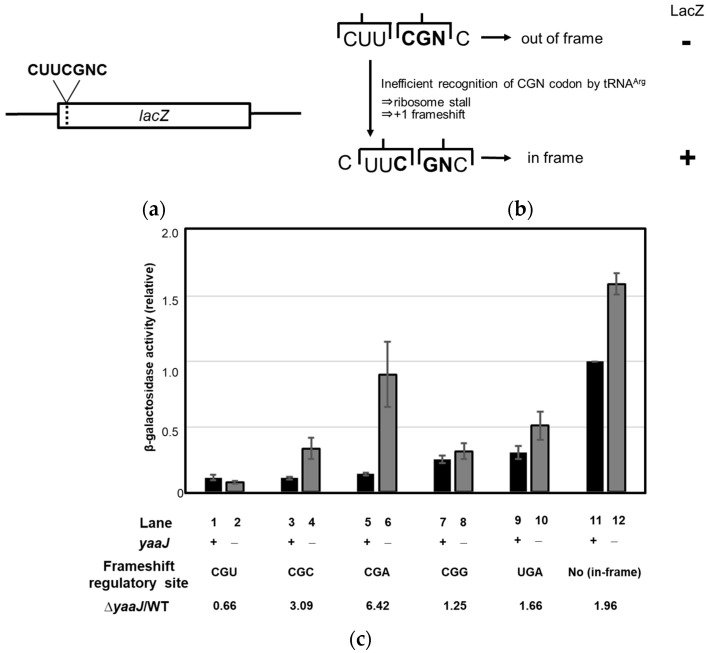
The frameshift-dependent lacZ/RF-2 reporter assay with or without I34 modification. (**a**) Schematic illustration of the *lacZ*/RF-2 reporter genes used in the β−gal assays showing the inserted nucleotides. (**b**) In the *lacZ*/RF-2 fusions, the regulatory UGA codon at the *E. coli* RF-2 frameshift site was replaced by the CGN codon, such that the generation of β−gal activity required frameshifting. In-phase translation (upper pathway) and +1 frameshift (lower pathway) were competing reactions at the regulatory frameshift site. (**c**) β−gal activity of *B. subtilis* strains with the *lacZ*/RF-2 gene with (+) or without (−) *yaaJ* (Table S2). The relative β−gal activity in the KUB10 strain versus the wild-type strain is indicated by KUB10/WT.

Even though the β−gal assay showed increased activity in the *yaaJ*-deletion mutant, I34 was still preferable for cell growth. Therefore, we hypothesized that I34 contributes to translational fidelity. To evaluate this possibility, a reporter assay based on the *E. coli* release factor 2 (RF-2) programmed frameshift mechanism [47,48] was performed in *B. subtilis* strains. In our analysis, the frameshift regulatory UGA codon in the *lacZ*/RF-2 fusion was replaced with a CGN codon, such that the generation of β−gal activity required frameshifting (Figure 5a,b). At this site, frameshifting and in-phase translation of the codon were competing reactions, and frameshift-dependent β−gal activity would then be inversely related to the rate of aminoacyl-tRNA selection. Thus, the frequency of frameshifting could be used to estimate the A site binding of tRNA at CGN codons.

The β−gal activities of both the original *lacZ* gene (with no frameshift site) and the gene with the UGA codon at the frameshift regulatory site in the KUB10 strain were higher than in the wild-type strain (lanes 9–12 in Figure 5c); these increases were the background as observed in Figure 4b. Insertion of the CGA codon at the regulatory site in the KUB10 strain showed a six-fold increase over the wild-type strain (lanes 5 and 6 in Figure 5c), indicating that the absence of I34 impeded A site selection and increased the frameshifting rate at the CGA codon. The second highest increase in frameshifting caused by the *yaaJ* deletion occurred when the CGC codon was at the regulatory site (lanes 3 and 4 in Figure 5c). This finding is explained by the chemical similarity between inosine and guanine, and also by the fact that unmodified A34 makes an unstable interaction with C(III) of the CGC codon in the A site. A slight decrease in the frameshifting rate in KUB10 cells was observed when the CGU codon was present at the regulatory site (lanes 1 and 2 in Figure 5c), suggesting that A34 is preferable for the stable recognition of U(III). The increase in β−gal activity of the KUB10 strain was slightly smaller when the CGG codon was contained at the regulatory site; the frameshifting at the CGG codons was repressed in the KUB10 strain. This implies that tRNA^Arg^(ACG) participates in decoding of the CGG codon, which is generally considered to be recognized exclusively by tRNA^Arg^(CCG).

Overall, these results indicate that I34 of tRNA^Arg^ ensures a smooth and stable interaction with CGN codons on the A site and that I34 contributes to the maintenance of the decoding fidelity, especially at the CGA codons in *B. subtilis*.

- tRNA^Arg^(CCG) is dispensable, and both tRNA^Arg^(ICG) and tRNA^Arg^(ACG) can each decode all four CGN codons in *B. subtilis*

The efficient translational activity independent of I34 suggests that the recognition of CGN codons is relatively relaxed in *B. subtilis*. We then attempted to construct a deletion mutant of tRNA^Arg^(CCG). tRNA^Arg^(CCG) encoded by a single gene; *trnQ*-*Arg(CCG)* was replaced with the antibiotic-resistance gene (Figure S1b). The resulting *trnQ-Arg(CCG)*-deletion mutant strains (SOM1 and SOM3 in Table 1) that lack tRNA^Arg^(CCG), as confirmed by Northern blotting (Figure S3a), were viable. The growth of SOM1 in LB at 37 °C was slowed in the stationary phase (Figure 3c). The β−gal activities of original and 4 × CGG-*lacZ* in SOM1 (lanes 3 and 16 in Figure 4b) were almost identical to that in the wild-type strain (lanes 1 and 14 in Figure 4b), showing that tRNA^Arg^(CCG) is not essential even for the decoding of the CGG codon.

Some species of mycoplasma and plant chloroplasts lack tRNA^Arg^(CCG) genes [18,31,32,60,61]. In mycoplasmas, the wobble position of transcripts derived from the gene encoding tRNA^Arg^(ACG) remains partially unmodified and is likely to decode CGG codons in the cell; it recruits both I34 and A34 to decode CGN codons [31]. There would be the possibility of a similar situation in our *B. subtilis trnQ-Arg(CCG)*-deletion mutant if a fraction of tRNA^Arg^(ACG) remained unmodified at the wobble position of the anticodon. We thus determined the anticodon sequence of tRNA^Arg^(I/ACG) from SOM1 cells by RT-PCR and sequencing analysis. As a result, all clones analyzed harbored I34 but not A34 (Figure S2c), suggesting that mature tRNA^Arg^(I/ACG) is completely inosine-modified in SOM1, and thus tRNA^Arg^ with I34 is solely responsible for the decoding all four CGN codons even in the absence of tRNA^Arg^(CCG). We also constructed a double deletion mutant of tRNA^Arg^(CCG) and *yaaJ*, and the resulting strain (SOM2 in Table 1) that contains only tRNA^Arg^(ACG) was viable. SOM2 grew more slowly in LB at 37 °C relative to the KUB10 or SOM1 strains (Figure 3c), whereas the β−gal activity in SOM2 was still higher than that in the wild-type strain (lanes 1 and 4 in Figure 4b). SOM2 exhibited cold sensitivity at 28 °C and below in LB medium (unpublished data).

The above results showed that a single repertoire, tRNA^Arg^(ICG) or tRNA^Arg^(ACG) can decode all four CGN codons in *B. subtilis*, suggesting that non-canonical recognition of G(III) by A34 or I34, A(III) by A34, and C(III) by A34 are possible in vivo (Figure 2).

- Decoding of the CGN codon family is not accomplished by C34

For further analysis, we attempted to construct a deletion mutant of tRNA^Arg^(ACG), encoded by four copies of the gene in the *B. subtilis* genome: *trnB*-Arg(ACG), *trnE*-Arg(ACG), *trnI*-Arg(ACG) and *trnJ*-Arg(ACG). A mutant strain lacking three genes of tRNA^Arg^(ACG) was generated, but a mutant lacking all four gene copies could not be obtained even when tRNA^Arg^(CCG) was located under the strong promoter (unpublished data), suggesting that the existence of tRNA^Arg^(ICG) or tRNA^Arg^(ACG) is crucial for viability and that tRNA^Arg^(CCG) is unable to decode all four CGN codons. We cannot exclude the possibility that the mutant with complete deletion of the tRNA^Arg^(ACG) genes was not obtained due to technical difficulties. But it is more plausible that tRNA^Arg^(CCG) cannot recognize all four CGN codons in *B. subtilis*.

## 4. Discussion

We have determined that *B. subtilis yaaJ* encodes the tRNA-adenosine deaminase (TadA) for the I34 formation of tRNA^Arg^. Our results showed that *yaaJ* is completely dispensable and thus tRNA^Arg^(ACG) with unmodified A34 plays an adequate role during translation. Intriguingly, β−gal activity in the *yaaJ*-deletion mutant was not impaired but slightly higher than that in the wild-type strain. These observations contrast with that of *E. coli*, in which the downregulation of the expression of *tadA* caused growth inhibition and severe reduction in β−gal activity (unpublished data). The exact mechanism for the increases in the β−gal activity was not clarified in this study. A systematic analysis such as RNA-seq or proteosome analysis would provide a clue to better understand the effect of I34-deletion in tRNA^Arg^ on mRNA stability, codon recognition, and overall translational efficiency. Besides being responsible for decoding the CGG codon, tRNA^Arg^(CCG) is non-essential whether in the presence or absence of *yaaJ*; it was unexpected that a single species of tRNA^Arg^ with either anticodon ICG or ACG decodes all four CGN codons in vivo (Figure 2). On the other hand, the tRNA^Arg^(I/ACG)-deletion mutant could not be obtained, indicating that tRNA^Arg^(CCG) is not adequate for the non-discriminating recognition of CGN codons. These results indicate that I34 and A34 of tRNA^Arg^ have an intrinsic ability to recognize all four nucleotides at the wobble position of the codon. The gaps in the β−gal activity among CGN codons in each strain suggest some direct interactions between I/A34 and N(III) in their base pairings.

Non-discriminating and extended codon recognition in nature have been reported for unmodified U34- or A34-containing tRNAs derived from specific bacteria and organelles that contain compact genomes encoding a reduced set of tRNA repertoires or that show a biased codon usage in which the corresponding codons are rarely used [13,21,31,60,61]. For example, in plant chloroplasts, the unmodified U34 of tRNA^Gly^(UCC) can decode all four GGN codons in the absence of tRNA^Gly^(GCC), while tRNA^Gly^(GCC) cannot assume that role [62]. In the mitochondria of fungi and nematodes, tRNA^Arg^ with unmodified ACG anticodon is the only tRNA that recognizes the CGN codon family, whereas CGA codon is rarely used in fungi mitochondria [31,32,63]. For such decoding systems, the “super-wobbling” (also known as “four-way wobbling” or “hyper-wobbling”) hypothesis has been suggested as a possible mechanism. In super-wobbling, a single tRNA species with an unmodified U34 or A34 reads all four nucleotides in the third position of the codon [61,62,64,65,66]. This hypothesis may apply to the ability of *B. subtilis* tRNA^Arg^(I/ACG) to decode CGN codons. Considering the existing idea that the I34-G(III) pairing is nonproductive, it was unpredictable that CGN codons are efficiently recognized solely by tRNA^Arg^(ICG) in the absence of tRNA^Arg^(CCG). A previous study has reported the recognition of all four nucleotides at the wobble position of the codon by I34 in yeast cells; tRNA^Ser^(IGA), responsible for the UCU, UCC, and UCA codons encoding serine, also recognizes the artificial non-sense codons, ΨAA and ΨAG, to suppress [67]. Our result showed the first example of non-discriminating recognition by I34 in bacterial cells and supports such unexpected ability of accommodation by the ribosomal decoding center in vivo.

Each of the CGN codons in non-pathogenic *E. coli* is biasedly used and the CGA codon is rarely found in essential genes, including ribosomal protein genes (Table S3). In *B. subtilis*, bias in CGN codon usage is relatively mild, and the CGA codon appears in ribosomal protein genes with a frequency comparable to those of the other CGN codons. This tendency is observed in other codon families [54,63], and such unbiased codon usage may correlate with the non-discriminating decoding property of *B. subtilis*. Additionally, there are fewer species of tRNA responsible for four-codon families in *B. subtilis* than in *E. coli*. For example, in *B. subtilis*, only one tRNA^Pro^ with the anticodon UGG of which U34 is modified to mo5U (5-methoxyuridine) decodes all four CCN codons, whereas three anticodons with cmo5U34 (5-carboxymethoxyuridine), G34 and C34 are assigned in *E. coli* (Figure 1) [11,18,68]. We have constructed a mutant strain of *B. subtilis* in which most of the four-codon family is decoded by a single species of anticodon (unpublished data). Therefore, a limited anticodon repertoire suffices for translation in *B. subtilis*.

Why is the super-wobbling, I34-N(III) and A34 -N(III) possible in *B. subtilis*? Codon–anticodon interaction dominantly depends on the hydrogen bonds between bases but it also involves other interactions to fit the overall shape of base pairings into the Watson–Crick geometry [69,70,71,72,73,74]. It has been suggested that non-discriminating codon recognition results from a combination of factors including the structural context of tRNA, competition with other tRNAs, and the nature of the ribosome. Sequences outside the anticodon including nucleotides at positions 32, 33, 37, and 38 in the anticodon-loop [26,75,76,77], and nucleotides at positions 9 and 24 in the D-arm [78,79,80], have been known to contribute greatly to decoding capacity and efficiency. Several differences found in sequence and modifications in tRNA^Arg^(ICG) from *B. subtilis* and *E. coli* may affect the stabilization of unorthodox wobbling. However, the introduction of *B. subtilis* tRNA^Arg^(ICG), which contains recognition elements for *E. coli* arginyl-tRNA synthetase [81], could not suppress the essentiality of *tadA* in *E. coli* (unpublished data), implying that the difference in the decoding ability of those bacteria is not derived solely from the tRNA. Previous studies have also indicated the intrinsic difference between the Gram-positive and Gram-negative bacteria in the decoding property [82,83,84]. Identification of the characteristics underlying the decoding property of *B. subtilis* is expected.

While the inosine modification of *B. subtilis* tRNA^Arg^ was dispensable, it was still preferable for translational fidelity, growth, and competency; this study partly answered the basic question of why the inosine modification has been actually conserved in this microbe. I34 modification would have a physiological role under stress conditions. However, sporulation was not affected, whereas competency was significantly inhibited in the absence of *yaaJ.* These observations seem to contradict each other, and further investigations are required to understand the respective mechanism. It is also possible that *yaaJ* has unidentified substrates other than tRNA. Recently, inosine modifications in mRNA of non-essential genes have been found in *E. coli* [10]. Although inosine modifications in mRNA have not been reported in *B. subtilis*, mRNA editing may have physiological functions in such a soil bacterium to survive harsh environments.

## Figures and Tables

**Table 1:** *B. subtilis* strains used in this study.

Strain	Genotype (Characteristics)	Source or Reference
168	*trpC2* (Wild-type)	Laboratory stock
KUB10	Δ*yaaJ*::*cat trpC* (*yaaJ*-deletion)	This study
SOM1	Δ*trnQ-Arg(CCG)*::*cat trpC2* (*trnQ*-deletion)	This study
SOM2	Δ*yaaJ::tet* Δ*trnQ-Arg(CCG)*::*cat trpC2* (*yaaJ*, *trnQ*-double deletion)	This study
SOM3	Δ*trnQ-Arg(CCG)*::*spc trpC2* (*trnQ*-deletion)	This study

**Table 2:** Sporulation efficiency of *B. subtilis* wild-type and KUB10 strains.

Strain	Viable Bacteria Count (c.f.u. mL^−^)		Frequency (%)
	Total	Spore	
Wild-type (*trpC2*)	(2.30 ± 0.63) × 10^8^	(2.06 ± 0.23) × 10^8^	89.6
KUB10 (Δ*yaaJ trpC2*)	(1.55 ± 0.14) × 10^8^	(1.09 ± 0.87) × 10^8^	70.0

Colony formation on plate medium was counted. The values are the mean of three independent experiments (*p* < 0.05). Means ± SD are shown.

**Table 3:** Transformation efficiency of *B. subtilis* wild-type and KUB10 strains.

Strain	Viable Bacteria Count (c.f.u. mL^−^)		Transformation Frequency (%)
	Total	Transformant	
Wild-type (*trpC2*)	(4.36 ± 0.17) × 10^8^	(1.16 ± 0.30) × 10^5^	0.027
KUB10 (Δ*yaaJ trpC2*)	(4.01 ± 0.22) × 10^8^	(1.17 ± 0.01) × 10^3^	0.0003

Colony formation on plate medium was counted. The values are the mean of three independent experiments (*p* < 0.05). Means ± SD are shown.

## Supplementary Materials

The following supporting information can be downloaded at: https://www.mdpi.com/article/10.3390/genes14081515/s1, Figure S1: Genetic maps of the *B. subtilis yaaJ*- and *trnQ-Arg(CCG)*-deletion mutants; Figure S2: Detection of inosine modification of the *B. subtilis* tRNA^Arg^(I/ACG) by DNA sequencing analysis of RT-PCR products; Figure S3: Northern blot analysis of *B. subtilis* tRNA^Arg^(I/ACG) and tRNA^Arg^(CCG); Figure S4: Growth of *B. subtilis* strains under various culture conditions; Figure S5: X−gal plate assay of *srfA* or *comK*-induced *lacZ* translation; Figure S6: Real-time PCR analysis of β−gal mRNA in wild-type and KUB10 strains; Table S1: Primers and probes used in this study; Table S2: *B. subtilis* strains used in β−gal assay; Table S3: Codon usage in *B. subtilis* and *E. coli*. References [11,56,63,85] are cited in the supplementary materials.
