# Supplementary material for: yaaJ, the tRNA-Specific Adenosine Deaminase, Is Dispensable in Bacillus subtilis
Source: Genes (Basel). 2023 Jul 25;14(8):1515. doi: 10.3390/genes14081515 (PMC10454642; doi:10.3390/genes14081515)
Supplement: Supplementary file 1 [file genes-14-01515-s001.zip › genes-2474759-supplementary.pdf]

genes

MDPI

Supplementary Materials:

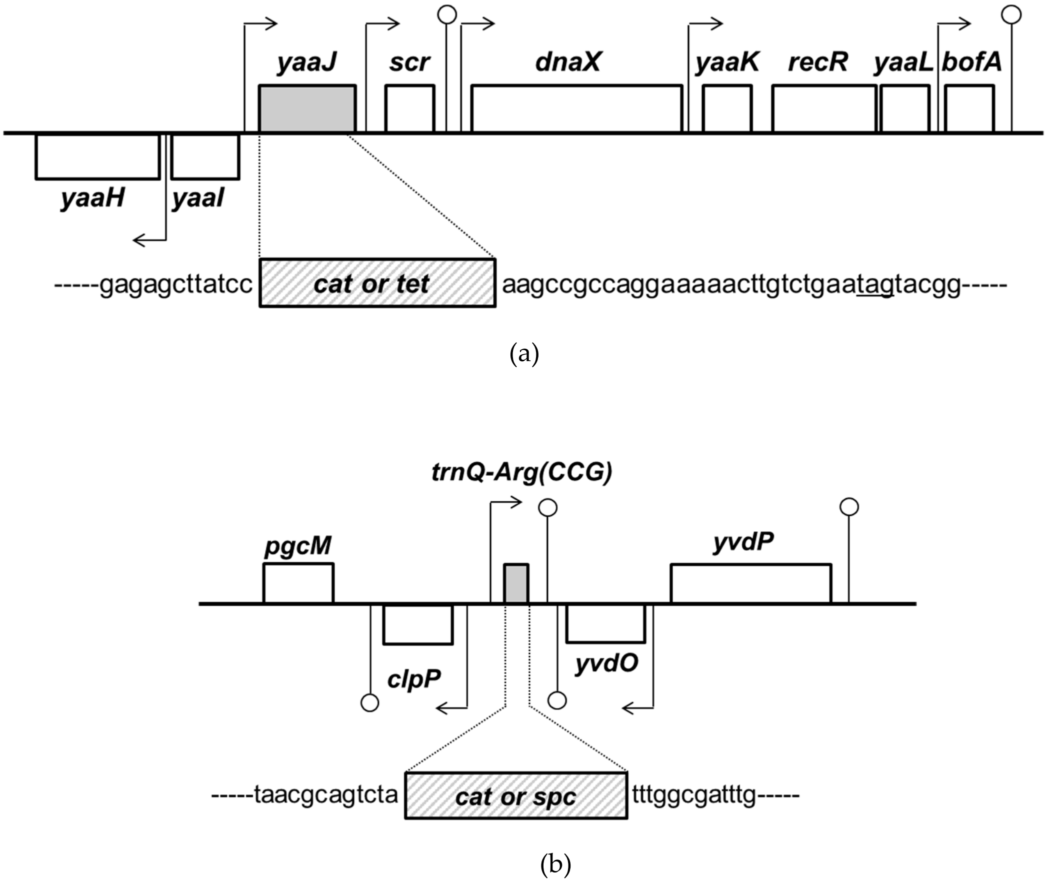

Figure S1. Genetic maps of the *B. subtilis yaaJ*- and *trnQ-Arg(CCG)*-deletion mutants.
Detailed protocols for the construction of mutants are described in the Materials and Methods. Genes encoding for (a) *yaaJ* and (b) *trnQ-Arg(CCG)* genes are shown by gray boxes. The predicted promoters and the terminators are shown by bars with arrows and circles, respectively. The junction points created by the introduction of the antibiotic-resistant gene are shown by the dotted lines. The stop codon of *yaaJ* is underlined.

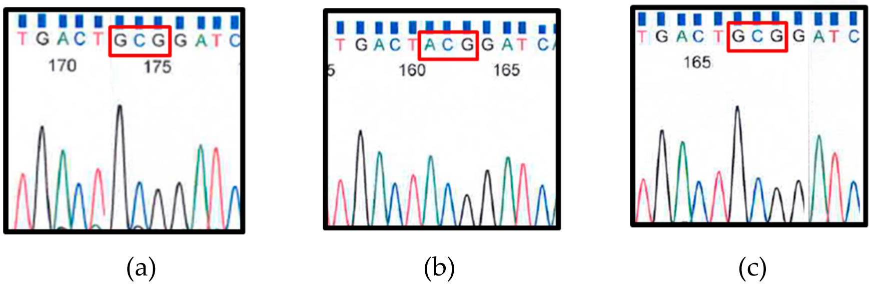

Figure S2. Detection of inosine modification in the anticodon of tRNA^{Arg}(I/ACG) by DNA sequencing analysis of RT-PCR products.
Sequencing analyses of tRNA^{Arg}(I/ACG) from (a) the wild-type (strain 168), (b) the *yaaJ*-deletion mutant (KUB10), and (c) the *trnQ-Arg(CCG)*-deletion mutant (SOM1) strains. The nucleotides of the anticodon are indicated. 5'- and RT'-primers used for RT-PCR are listed in Fig. S1.

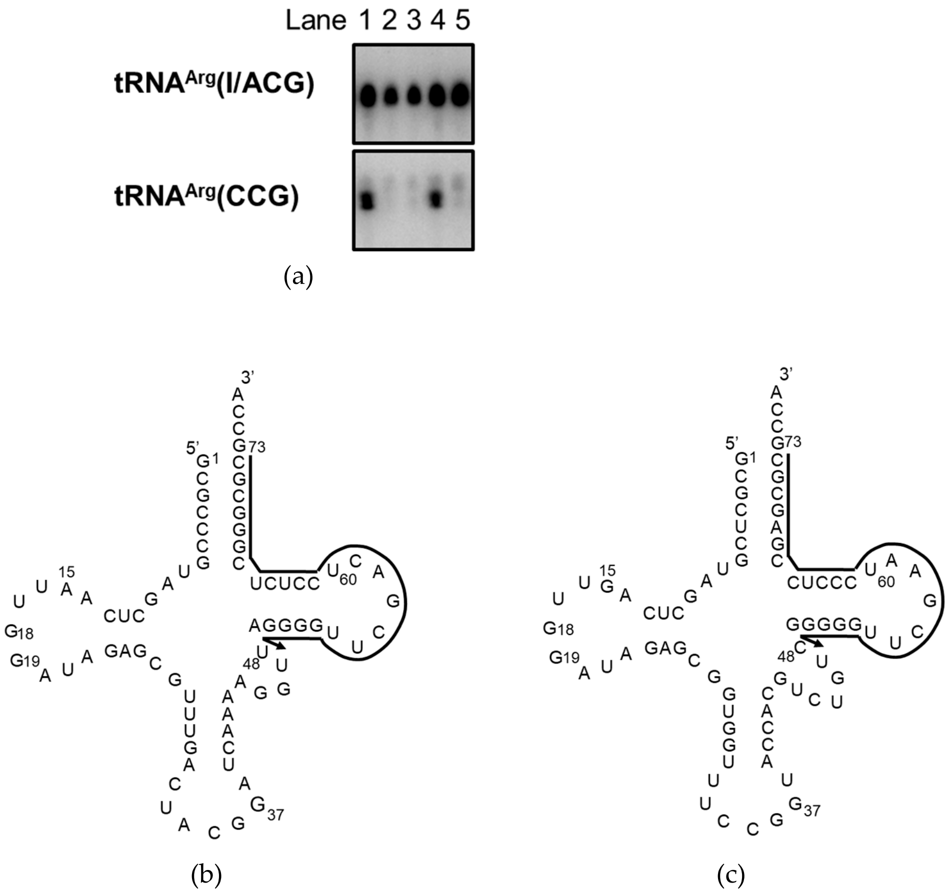

Figure S3. Northern blot analysis of *B. subtilis* tRNAArg(I/ACG) and tRNAArg(CCG).
(a) The expression of tRNA^{Arg}(I/ACG) and tRNA^{Arg}(CCG) was detected by probes complementary to the 3'-half of each tRNA, as indicated in (b) and (c). Crude RNA from the wild-type (strain 168 in lane 1), *trnQ-Arg(CCG)*-deletion mutant (SOM1 and SOM3 in lanes 2 and 3, respectively), *yaaJ*-deletion mutant (KUB10 in lane 4), and the double-deletion mutant of tRNA^{Arg}(CCG) and *yaaJ* (SOM2 in lane 5) strains. The numbering of the tRNA positions follows the reference [1].

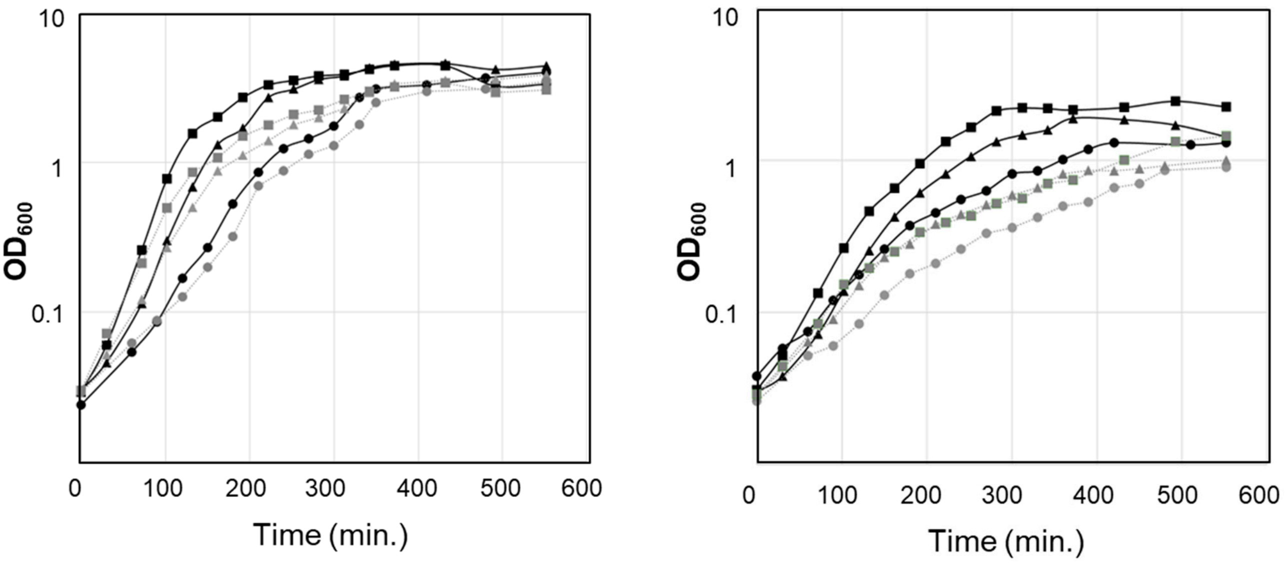

(a) (b)

Figure S4. Growth of *B. subtilis* strains under various culture conditions.
Growth of the wild-type (strain 168; solid black line) and the *yaaJ*-deletion mutant (KUB10; dashed gray line) in (a) the rich (LB) and (b) poor medium (CSM [2]) at 30°C (circles), 37°C (triangles) and 45°C (squares).

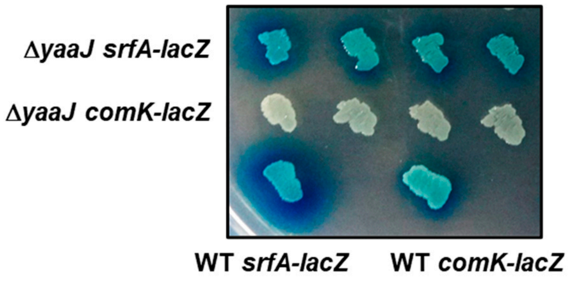

Figure S5. X−gal plate assay of *srfA* or *comK*-induced *lacZ* translation.
Wild-type (strain 168, bottom) and *yaaJ*-deletion mutant strains (KUB10, top and middle, 4 clones each) with the *srfA-lacZ* or *comK-lacZ* [3] in which the regulatory region and the first six codons (not arginine codons) of *comK* or *srfA* were fused to the N-terminus of *lacZ* were grown on the CI medium containing 40 μg/ml X−gal at 37°C.

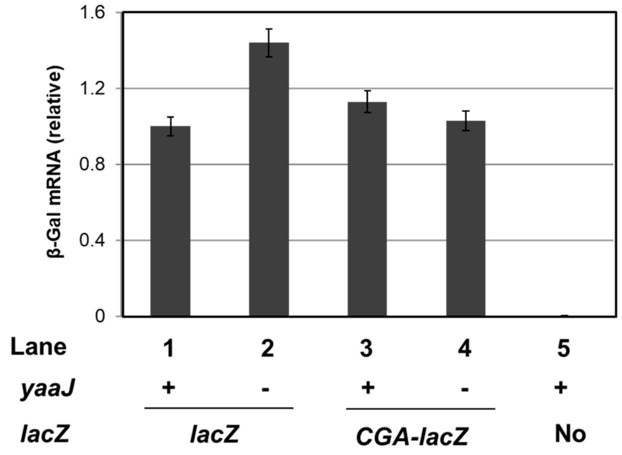

Figure S6. Real-time PCR analysis of β−gal mRNA in wild-type and KUB10 strains.
The relative amount of unmodified and tandem CGA-containing β−gal mRNA in the wild-type (strain 168) and *yaaJ*-deletion mutant (KUB10) strains are shown.

Table S1. Primers and probes used in this study.

Primer name	Sequences (5′→3′)	Relevant materials
yaaJ up5'-1	CATTCTCCATCATGGTTAAGGCGA	KUB10
yaaJ up3'-1	GCCTCCTAAATTTTTATCTAAAGTGGGAT AAGCTCTCTTTTCAAAGTT	KUB10
yaaJ dwn5'-1	TAATGACTGGCTTTTATAATATGAGAAGC CGCCAGGAAAAACTTGTCTG	KUB10
yaaJ dwn3'	TTTGTAATGTGTTCTTGTCCGACC	KUB10, SOM2
yaaJ up5'-2	GTATTGAACTGCCGGGCGATG	SOM2
yaaJ up3'-2	AACAATATGGCCCGTTTGTTGAACTGGAT AAGCTCTCTTTTCAA	SOM2
yaaJ dwn5'-2	CTCAAAGGGATTTCTAAATCGTTAAAAG CCGCCAGGAAAAACTTGTCTG	SOM2
trnQ-Arg(CCG)up5′-p	CGCCGGCTGCAAGCAGGAACGCGC	SOM1-3
trnQ-Arg(CCG)up3′p-1	GCCTCCTAAATTTTTATCTAAAGTG TAGACTGCGTTATGAGAACGTCAG	SOM1, SOM3
trnQ-Arg(CCG)dwn5′p-1	TAATGACTGGCTTTTATAATATGAGTTTG GCGATTTGCCGCTCTTGTCT	SOM1, SOM3
trnQ-Arg(CCG)dwn3′-p	GAACAGGTGAAGCCCCGACTCGGT	SOM1-3
trnQ-Arg(CCG)up3′p-2	CATGTATTCACGAACGAAAATCGATTAG ACTGCGTTATGAGAACGTCAG	SOM2
trnQ-Arg(CCG)dwn5′p-2	AAATTGAAAAAATGGTGGAAACACTTTG GCGATTTGCCGCTCTTGTCT	SOM3
cat5'p	CACTT TAGATAAAAA TTTAGGAGGC	KUB10, SOM1, SOM2
cat3'p	TTATAAAAGCCAGTCATTAGGCCTA	KUB10, SOM1, SOM2
spc5'p	ATCGATTTTCGTTCGTGAATACATG	SOM3
spc3'p	GTGTTTCCACCATTTTTTCAATTT	SOM3
tet5'p	AGTTCAACAAACGGGCCATA	SOM2
tet3'p	TTAACGATTTAGAAATCCCT	SOM2
lacZ sense	TCGAGAAAGGAGGTGAACTACTATGAAG CTTGGC	pTOM20c
lacZ antisense	TCGAGCCAAGCTTCATAGTAGTTCACCTC CTTTC	pTOM20c
lacZ CGU sense	TCGAGAAAGGAGGTGAACTACTATGCGT CGTAAGCTTCGTCGTGGC	pTOM21
lacZ CGU antisense	TCAGGCCACGACGAAGCTTACGACGCAT AGTAGTTCACCTCCTTTC	pTOM21
lacZ CGC sense	TCGAGAAAGGAGGTGAACTACTATGCGC CGCAAGCTTCGCCGCGGC	pTOM22
lacZ CGC antisense	TCGAGCCGCGGCGAAGCTTGCGGCGCAT AGTAGTTCACCTCCTTTC	pTOM22
lacZ CGA sense	TCGAGAAAGGAGGTGAACTACTAATGCG ACGAAAGCTTCGACGAGGC	pTOM23
lacZ CGA antisense	TCGAGCCTCGTCGAAGCTTTCGTCGCATA GTAGTTCACCTCCTTTC	pTOM23

lacZ CGG sense	TCGAGAAAGGAGGTGAACTACTATGCGG CGGAAGCTTCGGCGGGGC	pTOM24
lacZ CGG antisense	TCGAGCCCCGCCGAAGCTTCCGCCGCAT AGTAGTTCACCTCCTTTC	pTOM24
lacZ AGA sense	TCGAGAAAGGAGGTGAACTACTAATGAG AAGAAAGCTTAGAAGAGGC	pTOM25
lacZ AGA antisense	TCGAGCCTCTTCTAAGCTTTCTTCTCATA GTAGTTCACCTCCTTTC	pTOM25
lacZ GCU sense	TCGAGAAAGGAGGTGAACTACTAATGGC TGCTAAGCTTGCTGCTGGC	pTOM26
lacZ GCU antisense	TCGAGCCAGCAGCAAGCTTAGCAGCCAT AGTAGTTCACCTCCTTTC	pTOM26
RF sense (up)	GGCTCGAGAAAGGTGGTGAACTACTATG ACCATGATTACG	pKUB20,21,22,23, 24,25
RF sense (center)	ATGACCATGATTACGCCACCGCGGTGTA CATTTAGGGGGTCTCTT	pKUB20,21,22,23, 24,25
RF CGU (down)	GGCTCGAGCCACTAGTTGTACAGAGGAC GAAGAGACCCCCTA	pKUB20
RF CGC (down)	GGCTCGAGCCACTAGTTGTACAGAGGGC GAAGAGACCCCCTA	pKUB21
RF CGA (down)	GGCTCGAGCCACTAGTTGTACAGAGGTC GAAGAGACCCCCTA	pKUB22
RF CGG (down)	GGCTCGAGCCACTAGTTGTACAGAGGCC GAAGAGACCCCCTA	pKUB23
RF UGA (down)	GGCTCGAGCCACTAGTTGTACAGAGGTC AAAGAGACCCCCTA	pKUB24
RF no (nonframe)	GGCTCGAGGCACTAGTTGTACAGAGGTC AAGAGACCCCCTA	pKUB25
Arg(I/ACG)5′-p	GCGCCCGTAGCTCAATTG	RT-PCR primers for $tRNA^{Arg}$(I/ACG)
Arg(I/ACG)RT-p	CGAACCCCTAACCTTTTGATCC	RT-PCR primers for $tRNA^{Arg}$(I/ACG)
Arg(I/ACG)i-probe	CGCGCCCGAGAGGAGTCGAACCCCTAAC CT	Probe for isolation of $tRNA^{Arg}$(I/ACG)
Arg(I/ACG)n-probe	GCGCCCGAGAGGAGTCGAACCCCTA	Probe for $tRNA^{Arg}$(I/ACG)
trnQ-Arg(CCG)n-probe	GCGCTCGGAGGGATTCGAACCCCCG	Probe for $tRNA^{Arg}$(CCG)
Bs16S5'-p	AACCAGAAAGCCACGGCTAA	qPCR primer for 16S rRNA
Bs16S3'-p	GGACAACGCTTGCCACCTA	qPCR primer for 16S rRNA
lacZ5'-p	ATCAGGATATGTGGCGGATGA	qPCR primer for lacZ
lacZ3'-p	TGATTTGTGTAGTCGGTTTATGCA	qPCR primer for lacZ

Table S2. *B. subtilis* strains used in β-gal assay. Strains were used as follows: KUB11-KB32 for β-Galactosidase assay, KUB33-44 frameshift assay, and DFK1-2, SK149-150 for X-gal plate assay of *srfA* or *comK*-induced *lacZ* translation. All strains were constructed in this study.

Strain	Genotype (characteristics)
KUB11	*aprE::Pspac-lacZ trpC2* (Wild-type with *lacZ*)
KUB12	*aprE::Pspac-4×CGU-lacZ trpC2* (Wild-type with *4×CGU lacZ*)
KUB13	*aprE::Pspac-4×CGC-lacZ trpC2* (Wild-type with *4×CGC lacZ*)
KUB14	*aprE::Pspac-4×CGA-lacZ trpC2* (Wild-type with *4×CGA lacZ*)
KUB15	*aprE::Pspac-4×CGG-lacZ trpC2* (Wild-type with *4×CGG lacZ*)
KUB16	*aprE::Pspac-4×AGA-lacZ trpC2* (Wild-type with *4×AGA lacZ*)
KUB17	*aprE::Pspac-4×AGA-lacZ trpC2* (Wild-type with *4×GCU lacZ*)
KUB18	*aprE::Pspac-lacZ ΔyaaJ::cat trpC2* (*ΔyaaJ* with *lacZ*)
KUB19	*aprE::Pspac-4×GCU-lacZ ΔyaaJ::cat trpC2* (*ΔyaaJ* with *4×GCU lacZ*)
KUB20	*aprE::Pspac-4×GCC-lacZ ΔyaaJ::cat trpC2* (*ΔyaaJ* with *4×GCC lacZ*)
KUB21	*aprE::Pspac-4×GCA-lacZ ΔyaaJ::cat trpC2* (*ΔyaaJ* with *4×GCA lacZ*)
KUB22	*aprE::Pspac-4×GCG-lacZ ΔyaaJ::cat trpC2* (*ΔyaaJ* with *4×GCG lacZ*)
KUB23	*aprE::Pspac-4×GCG-lacZ ΔyaaJ::cat trpC2* (*ΔyaaJ* with *4×AGA lacZ*)
KUB24	*aprE::Pspac-4×GCG-lacZ ΔyaaJ::cat trpC2* (*ΔyaaJ* with *4×GCU lacZ*)
KUB25	*aprE::Pspac-lacZ trnQ-Arg(CCG)::cat trpC2* (*ΔArg(CCG)* with *lacZ*)
KUB26	*aprE::Pspac-4×GCU-lacZ trnQ-Arg(CCG)::cat trpC2* (*ΔArg(CCG)* with *4×GCU lacZ*)
KUB27	*aprE::Pspac-4×GCC-lacZ trnQ-Arg(CCG)::cat trpC2* (*ΔArg(CCG)* with *4×GCC lacZ*)
KUB28	*aprE::Pspac-4×GCA-lacZ trnQ-Arg(CCG)::cat trpC2* (*ΔArg(CCG)* with *4×GCA lacZ*)
KUB29	*aprE::Pspac-4×GCG-lacZ trnQ-Arg(CCG)::cat trpC2* (*ΔArg(CCG)* with *4×GCG lacZ*)
KUB30	*aprE::Pspac-4×GCG-lacZ trnQ-Arg(CCG)::cat trpC2* (*ΔArg(CCG)* with *4×AGA lacZ*)
KUB31	*aprE::Pspac-4×GCG-lacZ trnQ-Arg(CCG)::cat trpC2* (*ΔArg(CCG)* with *4×GCU lacZ*)
KUB32	*aprE::Pspac-lacZ ΔyaaJ::tetΔtrnQ-Arg(CCG)::cat trpC2* (*ΔyaaJΔArg(CCG)* with *lacZ*)
KUB33	*aprE::Pspac-CGUfs-lacZ trpC2* (Wild-type with *CGU-at-fs-lacZ*)
KUB34	*aprE::Pspac-CGCfs-lacZ trpC2* (Wild-type with *CGC-at-fs-lacZ*)
KUB35	*aprE::Pspac-CGAfs-lacZ trpC2* (Wild-type with *4×CGA-at-fs-lacZ*)
KUB36	*aprE::Pspac-CGGfs-lacZ trpC2* (Wild-type with *4×CGG-at-fs-lacZ*)
KUB37	*aprE::Pspac-AGAfs-lacZ trpC2* (Wild-type with *4×UGA-at-fs-lacZ*)
KUB38	*aprE::Pspac-if-lacZ trpC2* (Wild-type with in-frame *lacZ*)
KUB39	*aprE::Pspac-CGUfs-lacZ ΔyaaJ::cat trpC2* (*ΔyaaJ* with *CGU-at-fs-lacZ*)
KUB40	*aprE::Pspac-CGCfs-lacZ ΔyaaJ::cat trpC2* (*ΔyaaJ* with *CGC-at-fs-lacZ*)
KUB41	*aprE::Pspac-CGAfs-lacZ ΔyaaJ::cat trpC2* (*ΔyaaJ* with *4×CGA-at-fs-lacZ*)
KUB42	*aprE::Pspac-CGGfs-lacZ ΔyaaJ::cat trpC2* (*ΔyaaJ* with *4×CGG-at-fs-lacZ*)
KUB43	*aprE::Pspac-AGAfs-lacZ ΔyaaJ::cat trpC2* (*ΔyaaJ* with *4×UGA-at-fs-lacZ*)
KUB44	*aprE::Pspac-if-lacZ ΔyaaJ::cat trpC2* (*ΔyaaJ* with in-frame *lacZ*)
DF1	*amyE::srfA-lacZ trpC2* (Wild-type with *comK-lacZ*)
DF2	*amyE::comK-lacZ trpC2* (Wild-type with *srfA-lacZ*)
SK149	*amyE::srfA-lacZ ΔyaaJ::tet trpC2* (*ΔyaaJ* with *srfA-lacZ*)
SK150	*amyE::comK-lacZ ΔyaaJ::tet trpC2* (*ΔyaaJ* with *comK-lacZ*)

Table S3. Codon usage in *B. subtilis* and *E. coli*.
The number and relative synonymous codon usage (RSCU) of 6 arginine codons for all genes in *B. subtilis* and *E. coli* are shown [4]. RSCU is calculated as the ratio of the observed frequency of a codon to the expected frequency under the assumption of equal usage between synonymous codons for the same amino acids.

	B. subtilis 168		*E. coli* K12	
Codon	Count	RSCU	Count	RSCU
CGU	9133	1.091	28149	2.296
CGC	10397	1.241	29560	2.411
CGA	4962	0.592	4664	0.38
CGG	7833	0.935	7119	0.581
AGA	13221	1.579	2625	0.214
AGG	4703	0.562	1457	0.119

References

1. Jühling, F.; Mörl, M.; Hartmann, R.K.; Sprinzl, M.; Stadler, P.F.; Pütz, J. tRNAdb 2009: Compilation of tRNA sequences and tRNA genes. *Nucleic Acids Res.* **2009,** *37,* D159–D162. doi: 10.1093/nar/gkn772.
2. Murayama, R.; Akanuma, G.; Makino, Y.; Nanamiya, H.; Kawamura, F. Spontaneous transformation and its use for genetic mapping in *Bacillus subtilis*. *Biosci. Biotechnol. Biochem.* **2004,** *68,* 1672-1680. doi: 10.1271/bbb.68.1672.
3. Kong, J. H.; Dubnau, D. The regulation of competence transcription factor synthesis constitutes a critical control point in the regulation of competence in *Bacillus subtilis*. *J. Bacteriol.* **1994,** *176*, 5753-5761. doi: 10.1128/jb.176.18.5753-5761.
4. Subramanian, K.; Payne, B.; Feyertag, F.; Alvarez-Ponce, D. The codon statistics database: A database of codon usage bias. *Mol. Biol. Evol.* **2022**, *39,* msac157. doi: 10.1093/molbev/msac157.
